# Supplementary material for: Barriers to and enablers of quality improvement in primary health care in low- and middle-income countries: A systematic review
Source: PLOS Glob Public Health. 2024 Jan 18;4(1):e0002756. doi: 10.1371/journal.pgph.0002756 (PMC10796071; doi:10.1371/journal.pgph.0002756)
Supplement: S1 Table — (PDF) [file pgph.0002756.s002.pdf]

S1 Table. Key words used when searching databases and websites

Sample size	Phenomenon of interest	Design of studies	Evaluation	Research type
Health worker (all cadres & levels, stakeholders)	Quality improvement in primary health care	Qualitative OR “Mixed Methods”	Barrier* OR limitation* OR constraint* OR enabler* OR promoter* OR facilitator* OR Attitude* OR belief* OR practice* OR knowledge* OR perception* OR perspective* OR behaviour* OR culture OR motivation OR beliefs OR value* OR factor*	Observation OR Interview OR “Focus Group” OR Survey OR Questionnaire OR “Case Study” OR KII OR IDI OR FGD OR “Participant observation” OR OR “Group Interview”
“Health managers” OR<br>“Quality improvement team” OR<br>“Quality improvement committee*” OR<br>“Health service provider” OR<br>“Primary care team” OR<br>“Primary care physicians” OR<br>“Health cent* workers” OR<br>“Dispensary worker*” OR<br>“Health post worker*” OR	(“Health care quality improvement” OR “Quality Improvement” OR) AND (Primary Health Care” OR “Essential health care” OR “Basic Health Care” OR QI OR “Quality enhancement” OR (“Curative OR Rehabilitative OR Prevent* OR Promot* AND health)			

“Community health worker*” OR<br><br>“Primary care network” OR Primary<br><br>Health care network” OR PCN				
