# Supplementary material for: Barriers to and enablers of quality improvement in primary health care in low- and middle-income countries: A systematic review
Source: PLOS Glob Public Health. 2024 Jan 18;4(1):e0002756. doi: 10.1371/journal.pgph.0002756 (PMC10796071; doi:10.1371/journal.pgph.0002756)
Supplement: S4 Table — (PDF) [file pgph.0002756.s005.pdf]

S4 Table. Analytical framework with themes and sub-themes

Theme	Sub-themes	Study - country
Microsystem: individual health worker motivation for quality improvement	*Enablers:*<br>-developing empathy and better communication with clients<br>-Intrinsic motivation i.e., job satisfaction from participation in QI activities motivates health workers to put in more effort and strong desire to help one's own community<br>-increased familiarity with patient-centered care approaches, deeper connections between health worker and clients<br>-extrinsic motivation drawn from financial incentives and understanding rationale for QI<br>-strong culture of valuing data as a tool to drive improvements<br>-high level of technical and managerial proficiency promotes effective data collection, analysis, and use gained over time<br>-feeling empowered and competent after participating in training<br>-better understanding of roles and responsibilities in QI by health workers and increasing levels of comfort with QI tools	**Africa (Low-income):** Tibeihaho et al (2021) – Uganda; Kim et al (2019) - Uganda; Hutchinson et al (2021) - Uganda; Gage et al (2022) - Zimbabwe; Baker et al (2018) - Tanzania; Coulibaly et al (2020) - Mali; Lokossou et al (2019) - Benin; Stover et al (2014) - Ethiopia; Quaife et al (2021) - Ethiopia; Manzi et al (2014) - Rwanda; Werdenberg et al (2018) - Rwanda; Hounsou et al (2022), Benin<br><br>**Africa (Lower middle-income):** Giessler et al (2020) - Kenya; Eboreime et al (2018) - Nigeria; Eboreime et al (2019) - Nigeria;

	-personal motivation after observing changes due to QI and being thanked by clients/ patients<br>-regular review meeting to identify gaps and root causes, action planning to address gaps<br>-health workers inspired by committed health facility/district leaders and QI mentors<br>-health workers shift attitude to focus more on patient needs with desire to alleviate pain and suffering and reduce deaths<br>- health workers learn and embrace better ways of solving problems and become more systematic, working across disciplinary boundaries<br>-district managers' ability to use contextualized data for QI<br>-health workers like internal supervision for knowledge sharing and skills development<br>-QI intervention promotes transparency and stirs up healthy competition<br>-NGO-owned health facility worker's norms embrace accountability (performance-driven)<br>-embrace of personal sacrifice and effort to earn public praise for health workers<br>-growing dissatisfaction with poor state of service quality<br>-shared values such as cohesion, merit, individual responsibility, maintaining high standards of work	Olaniran et al (2022) Nigeria; Odusola et al (2016) - Nigeria<br><br>**Africa (Upper middle-income)**: Yapa et al (2022) - South Africa; Horwood et al (2023) - South Africa; Kinney et al (2022) – South Africa<br><br>**Asia (UMIC):** Limato et al. (2019) - Indonesia; Thekkur et al (2022) - Sri Lanka; Lall et al (2020) - India; Werner et al (2021) - Tajikistan; Schuele and MacDougall (2022) - Papua New Guinea<br><br>**Americas (LIC):** Demes et al (2021) - Haiti

	Barriers:<br>-no spare time for health worker to attend QI meetings due to clinical duties<br>-financial disincentives lead to frustration and waning interest in QI<br>-overlapping data systems increase distract from provision of care to patients<br>-public (government-owned) health facilities reject QI focused on greater transparency and accountability due ingrained<br>-sensing despair and easily giving up on QI initiatives<br>-self-efficacy is limited when more manager approvals are needed to carry out work tasks than are necessary and staff feel unskilled (technical/clinical areas and ICT)<br>-tasks perceived to be time-consuming lower health worker confidence<br>-unsupportive colleagues at the workplace<br>-lack of recognition of presumed hard work<br>-negative culture that rejects use of care delivery checklists and declines referrals even when indicated	**Americas (UMIC):** Pesec et al (2021) - Costa Rica<br><br>**Multi-country:** Djellouli et al (2016) - Malawi, Kenya, Burkina Faso and Mozambique; Kinney et al (2020) -Tanzania, Nigeria, Rwanda, Zimbabwe; Sukums et al (2015) - Tanzania and Ghana

QI Intervention Attributes	*Enablers:*<br>-QI project implementation perceived to be effective i.e., positive outcomes for patients and health workers (implementers) also acquire new skills and knowledge<br>-QI project is considered feasible, timely and well aligned local priorities<br>-health workers see a high degree of fit between QI package, their job responsibilities and practice expectations<br>-health workers see a relative advantage of QI package versus current practice<br>-QI intervention adapted and pre-tested to suit local implementation conditions<br>-Intervention is focused on a specific problem, is not too general and does not try to address too many things at once<br>-participants feel confident continuing with QI even post-intervention period<br>-QI intervention can be scaled up to other areas, health facilities, or health workers in need<br>-QI project details clear management structures and does not ignore or assume this<br>-project design fosters collaboration among diverse workers and even clients<br>-Intervention design incorporates and complements participants/health system’s values	**Africa (Low-income):** Hounsou et al (2022) - Benin; Coulibaly et al (2020) - Mali; Gage et al (2022) - Zimbabwe; Stover et al (2014) - Ethiopia; Quaife et al (2021) - Ethiopia; Ayele et al (2019) - Ethiopia; Tiruneh et al (2020) - Ethiopia; Tibeihaho et al (2021) - Uganda; Kim et al (2019) - Uganda; Hutchinson et al. (2021) - Uganda; Werdenberg et al (2018) - Rwanda; Umunyana et al (2020) - Rwanda<br><br>**Africa (Lower middle-income):** Giessler et al (2020) - Kenya; Eboreime et al (2018) - Nigeria; Eboreime et al (2019) - Nigeria; Olaniran et al (2022) – Nigeria; Tancred et al (2016) - Tanzania; Jaribu et al (2017) - Tanzania; Tancred et al (2018) - Tanzania;

	-QI intervention design makes provision for long-term work to sustain changes and its costs do not overwhelm the systems' resource capacity<br>-Intervention adopts small incremental changes informed by feedback mechanisms rather than big rapid leaps<br>-intervention design incorporates client preferences, not only health workers' ideas<br><br>*<u>Barriers:</u>*<br>-QI project does not lead to any observable improvements<br>-QI implementation plans do not attain targeted levels of penetration (low does/reach)<br>-QI intervention package is hard to understand, not easy to translate into tangible action points, and perceived as not user-friendly<br>-lack of clear implementation plan for QI intervention<br>-QI intervention is difficult to integrate in routine practice and or requires substantial modifications to workflows and additional new skills<br>-in technology-driven QI, perception that the new approach is inflexible or rigid	Pallangyo et al (2018) - Tanzania; Baker et al (2018) - Tanzania;<br><br>**Africa (Upper middle-income)**: Basenero et al (2022 - Namibia; Yapa et al (2022) - South Africa; Mantell et al (2022) - South Africa; Mutambo et al (2020) - South Africa; Kinney et al (2022) – South Africa; Horwood et al (2023) - South Africa<br><br>**Asia (Upper middle-income)**: Lall et al (2020) - India; Schierhout et al (2021) - India; Werner et al (2021) - Tajikistan; Schuele and MacDougall (2022) - Papua New Guinea; Thekkur et al (2022) - Sri Lanka; Limato et al (2019) - Indonesia

	-QI intervention has perceived negative unintended or unanticipated consequences e.g., creates more administrative burden on already overstretched health staff<br>-Intervention does not allow implementers (who see it as alien or imposed upon them) to make or suggest adaptations<br>-intervention package does not envisage nor address other contextual and systems barriers to its successful implementation (focus on short term technical fixes and does not address or consider structural bottlenecks)<br>-QI intervention does not build on existing initiatives	**Americas (Low-income):** Demes et al (2021) - Haiti<br><br>**Americas (Upper middle-income)**: Pesec et al (2021) - Costa Rica<br><br>**Multi-country:** Sukums et al (2015) - Tanzania and Ghana; Kinney et al (2020) - Tanzania, Nigeria, Rwanda, Zimbabwe; Chandani et al 2017) - Rwanda and Malawi; Djellouli et al (2016) - Malawi, Kenya, Burkina Faso and Mozambique

Organization and Team implementing QI	*<u>Enablers</u>:*<br>-managers and team members agree to additional responsibilities<br>-seniour leaders embrace and support QI<br>-experienced subject matter experts drive change<br>-collegiality or team spirit in decision making beginning from the start of QI project<br>-presence of QI champions in the team<br>-balance between top-down and bottom-up approaches in decision making<br>-team enthusiastic and (publicly) committed<br>-everyone involved with diverse inputs<br>-a quality culture with shared values, attitudes and behaviour of everybody becomes embedded in the organisation's fabric e.g., regular data analysis, action and improvement cycles<br>-organization allocates budget, avails resources for QI<br>-physicians take lead, build others' skills<br>-trained team members report back, share knowledge and skills with colleagues e.g., on Plan-Do-Study-Act cycles and problem-solving	**Africa (Low income):**<br>Coulibaly et al (2020) - Mali<br>Nahimana et al (2021) - Rwanda; Umunyana et al (2020) - Rwanda; Stover et al (2014) - Ethiopia<br><br>**Africa (Lower middle-income)**: Eboreime et al (2018) – Nigeria; Baker et al (2018) - Tanzania; Pallangyo et al (20180 - Tanzania<br><br>**Africa (Upper middle-income):** Kinney et al (2022) – South Africa; Mantell et al (2022) - South Africa; Yapa et al (2022) - South Africa; Horwood et al (2023) - South Africa

	-regular, positive feedback on QI project shared with stakeholders including good internal communication<br>-positive team experiences from successful legacy QI projects produce domino effect<br>-adequate team preparation before introduction of QI<br>-regular on-the-job training in addition to classroom sessions<br>-accreditation process inspires and supports drive to improve service quality<br><br>*Barriers:*<br>-frozen relationships between managers and frontline implementers<br>-organization does not own (rejects) new QI initiative<br>-team members lack knowledge or skills on QI approaches<br>-lack of clarity on QI stewardship and monitoring arrangements<br>-The 'missing middle' in decentralized settings (unsupportive district-level managers)<br>-concurrent similar QI programmes in the same organization bring confusion and uncertainty<br>-team neglects to include support (non-technical) staff	**Asia (Upper middle-income):** Schierhout et al (2021) - India; Limato et al (2019) - Indonesia; Schuele and MacDougall (2022) - Papua New Guinea; Werner et al (2021) - Tajikistan<br><br>**Americas (Low income)**: Demes et al (2021) - Haiti<br><br>**Americas (Upper middle-income):** none<br><br>**Multi-country:** Kinney et al (2020) - Rwanda, Tanzania, Zimbabwe, Nigeria; Chandani et al (2017) – Rwanda and Malawi

	-team leaders do not genuinely involved others in decisions<br><br>-weak leadership by government sees QI left to partners/donors<br><br>-one-off training for QI team norms<br><br>-QI focal persons wearing too many hats	
Health Systems Support and Capacity	*Enablers:*<br><br>-available staff with aligned job descriptions and incentives<br><br>-adequate, well designed physical space and infrastructure<br><br>-facilitative and supportive supervision<br><br>-regular follow up and mentorship<br><br>-silos and lack of integration<br><br>-provision of adequate supplies and commodities to deliver services<br><br>-strong patient referral<br><br>-participatory and data-driven QI activities<br><br>-data and reporting tools are revised to ensure one harmonized system of reports<br><br>*Barriers:*	**Africa (Low-income):** Manzi et al (2014) - Rwanda; Tayebwa et al (2020) - Rwanda; Nahimana et al (2021) - Rwanda; Umunyana et al (2020) - Rwanda; Werdenberg et al (2018) - Rwanda; Bradley et al (2012), Ethiopia; Stover et al (2014), Ethiopia; Ayele et al (2019), Ethiopia; Coulibali et al (2020) - Mali; Hounsou et al (2022) - Benin<br><br>**Africa (Lower middle-income):** Eboreime et al (2018) - Nigeria; Olaniran et al (2022) –

	-stockouts of drugs and supplies<br><br>- inadequate patient referral systems<br><br>-unpredictable follow up and punitive or unfocused supervision<br><br>-frequent staff leave of absence<br><br>-high staff turnover at health facility<br><br>-low numbers of health workers with high work loads<br><br>-poorly designed or inadequate space and infrastructure<br><br>-lack of equipment (ICT/data and medical devices)<br><br>-insufficient engagement of district level<br><br>-inadequate patient records system at the health facility level constrains service delivery	Nigeria; Baker et al (2018) - Tanzania; Pallangyo et al (2018) - Tanzania<br><br>**Africa (Upper middle-income):** Kinney et al (2022) – South Africa; Yapa et al (2022) - South Africa; Horwood et al (2023) - South Africa; Mantell et al (2022) - South Africa; Basenero et al (2022 - Namibia<br><br>**Asia (Upper middle-income):** Thekkur et al (2022) - Sri Lanka; Schierhout et al (2021) - India; Werner et al (2021) - Tajikistan; Limato et al (2019) - Indonesia<br><br>**Americas:** none

		**Multi-country:** Chandani et al (2017) - Rwanda and Malawi; Sukums et al (2015) - Tanzania and Ghana; Djellouli et al (2016) - Malawi, Kenya, Burkina Faso and Mozambique; Kinney et al (2020) - Rwanda, Tanzania, Zimbabwe and Nigeria
External environment and structural factors	*__Enablers:__*<br>-needed policies, plans, budgets and guidelines in place and conducive<br>-conducive financing and technical policies and guidelines<br>-high political visibility for QI intervention<br>-social norms encourage positive collaboration, problem solving and success<br>-strong political commitment for change<br><br>*__Barriers:__*<br>-difficult access to/for communities with poor road networks	**Africa (Low-income):** Lokossou et al (2019) - Benin; Coulibaly et al (2020) - Mali; Bradley et al (2012) - Ethiopia; Nahimana et al (2021) - Rwanda; Werdenberg et al (2018) - Rwanda<br><br>**Africa (Lower middle-income):** Olaniran et al (2022) - Nigeria

	-conflicts and insecurity, drought and famine<br>-bad political and socio-economic policies<br>-international and donor-led priority-setting<br>-PHC not prioritised - more focus on secondary and tertiary care by government and international agencies<br>-financial access barriers and poverty<br>-donor-driven priority setting<br>-larger health systems configuration e.g., employment conditions and administrative set up<br>-poor roads, energy & telecommunications infrastructure<br>- poor weather conditions<br>-disruptive onset of COVID-19 pandemic<br>-weak regulation and integration of private PHC service providers in health system<br>-weak collaboration and coordination between central and peripheral (local) government structures	**Africa (Upper middle-income):** Yapa et al (2022) - South Africa; Horwood et al (2023) - South Africa; Mantell et al (2022) - South Africa; Mutambo et al (2020) - South Africa; Kinney et al (2022) - South Africa<br><br>**Asia (Upper middle-income):** Werner et al (2021) - Tajikistan; Thekkur et al (2022) - Sri Lanka<br><br>**Americas:** none<br><br>**Multi-country:** Djellouli et al (2016) - Kenya, Malawi, Mozambique, Burkina Faso; Sukums et al (2015) - Tanzania and Ghana; Kinney et

		al (2020) - Rwanda, Tanzania, Zimbabwe, Nigeria
Execution of QI Intervention	*Enablers:*<br>-Implementers work collaboratively with community resource persons and civil society, draw upon local knowledge to tailor communication to clients and to effectively engage with communities<br>-champions are identified across all levels of the organization and system and take lead on modelling new roles in PHC while emphasizing collaborative working<br>-adequate numbers of implementers receive ongoing knowledge and practice updates from knowledgeable mentors and supervisors, and supervision/mentorship sessions embrace reflexivity and reflective practice.<br>-unconstrained communication makes use of multiple channels, provides avenue for (real-time) feedback and information sharing across all levels and types of QI stakeholders and facilitates decision-making<br>-including reminders in home-based records for patients where applicable<br>-re-designing clinic workflow, as needed, in a patient-centered manner	**Africa (Low-income):** Coulibaly et al (2020) - Mali; Hounsou et al (2022) - Benin; Stover et al (2014) - Ethiopia; Bradley et al (2012) - Ethiopia; Ayele et al (2019) - Ethiopia; Quaife et al (2021) - Ethiopia; Manzi et al (2014) - Rwanda; Werdenberg et al (2018) - Rwanda; Nahimana et al (2021) - Rwanda; Umunyana et al (2020) - Rwanda; Tayebwa et al (2020) - Rwanda; Hutchinson et al (2021) - Uganda<br><br>**Africa (Lower middle-income):** Eboreime et al (2018) - Nigeria; Olaniran et al (2022) – Nigeria; Jaribu et al (2016) - Tanzania; Pallangyo et al (2018) - Tanzania; Tancred et

	-stocks of key commodities are tracked and reported regularly<br><br>-results-oriented work plans are developed and executed participatorily<br><br>-QI implementation includes enhancements in documentation of care processes<br><br>-intervention is executed in incremental doses where subsequent sessions build on earlier ones in a responsive manner<br><br>-there is verification (monitoring) of whether QI activities are implemented in line with plans using data from PHC facilities<br><br>-influencers and blockers are identified and engaged during QI implementation<br><br>-QI training sessions are offered repeatedly to reach most implementers<br><br><br>*<u>Barriers:</u>*<br><br>-QI implementation does not consider availability of staff and competing tasks, leading to some health workers missing meetings and training sessions<br><br>-focus of intervention remains limited throughout implementation period, and not all planned aspects get rolled out. Late roll out of only a few aspects.<br><br>-clients keep off PHC facilities due to past negative experiences when seeking care	al (2018) - Tanzania; Baker et al (2018) - Tanzania<br><br><br>**Africa (Upper middle-income):** Basenero et al (2022 - Namibia; Yapa et al (2022) - South Africa; Mantell et al (2022) - South Africa; Mutambo et al (2020) - South Africa; Horwood et al (2023) - South Africa; Kinney et al (2022) - South Africa<br><br><br>**Asia (Upper middle-income):** Thekkur et al (2022) - Sri Lanka; Limato et al (2019) - Indonesia; Schierhout et al (2021) - India; Werner et al (2021) - Tajikistan<br><br><br>**Americas:** none

	-implementation plans considered over-ambitious and unrealistic<br>-limited training and supervision of health service providers create gaps in implementation<br>-community clients stay away due to low or non-involvement of local leaders and administrators exposing only a few clients to the QI intervention that targets them<br>-implementers withhold feedback from other stakeholders including communities contributing to mistrust, misperceptions, and constrained relationships<br>-lack of support supervision during QI implementation<br>-objectives of QI sessions are not discussed or shared widely<br>-limited risk communication and communities remain unaware of the need to shift behaviours and practices to healthier options promoted by QI intervention<br>-implementers do not keep track of the availability of drugs and other stocks<br>-implementation is skewed away from agreed plans to meet donor demands<br>-health workers do not practice new skills gained from QI for extended periods leading to decay of knowledge and skills<br>-users (in case of technology) experience delays when stuck and need support	**Multi-country:** Djellouli et al (2016) - Kenya, Malawi, Mozambique, Burkina Faso; Kinney et al (2020) - Tanzania, Nigeria, Rwanda, Zimbabwe; Chandani et al (2017) - Rwanda and Malawi; Sukums et al (2015) - Tanzania and Ghana
