# Supplementary material for: Barriers to and enablers of quality improvement in primary health care in low- and middle-income countries: A systematic review
Source: PLOS Glob Public Health. 2024 Jan 18;4(1):e0002756. doi: 10.1371/journal.pgph.0002756 (PMC10796071; doi:10.1371/journal.pgph.0002756)
Supplement: Attachment — Submitted filename: Response to Reviewers.docx [file pgph.0002756.s009.docx]

| **Editor/Peer Reviewer Comment** | **Author action/comment** |
| --- | --- |
| *Part A: Journal Requirements:* |  |
| Please provide separate figure files in .tif or .eps format only and remove any figures embedded in your manuscript file. Please also ensure all files are under our size limit of 10MB.  While revising your submission, please upload your figure files to the Preflight Analysis and Conversion Engine (PACE) digital diagnostic tool, <https://pacev2.apexcovantage.com/>. PACE helps ensure that figures meet PLOS requirements. To use PACE, you must first register as a user. Registration is free. Then, login and navigate to the UPLOAD tab, where you will find detailed instructions on how to use the tool. If you encounter any issues or have any questions when using PACE, please email PLOS at [figures@plos.org](mailto:figures@plos.org). **Please note that Supporting Information files do not need this step.** | No figures embedded in manuscript file. Figures converted to .tif using PACE.  Supplementary Information figures not converted as these are multi-page and excluded from the requirement. |
| *Part B: Reviewer #1* |  |
| Reviewer #1: The authors have worked on a very important topic which will help to demystify the challenging situation which faces countries (at national, sub-national, and facility level) in sustaining quality improvement initiatives. As the world marks the half-way in the race towards achieving the Sustainable Development Goals (SDGs) 2030; especially in the SDG 3 in which quality of services is an integral component of the target 3.8 on Universal Health Coverage (UHC), this manuscript is very useful. The paper is well conceptualized and written. However, there are minor areas which need some minor revisions (corrections/improvements) as explained below.  Title page  Key words: there is a repetition of the key word “primary health care”. Therefore, I suggest to the authors to delete one primary health care. | Deleted one “primary healthcare”. |
| 38 Introduction   Line 41: ADD the abbreviation LMICs in brackets as follows – countries (LMICs).   Line 47: replace “lower- and middle-income countries” with its abbreviation – LMICs.   Line 48: replace “lower- and middle-income countries (LMICs)” with its abbreviation – LMICs.   Line 58: replace “primary health care” with its abbreviation – PHC.   Line 62: replace “primary health care (PHC)” with its abbreviation – PHC.   Line 72: replace “quality improvement” with its abbreviation QI.   Lines 79-80: replace “quality improvement” with its abbreviation QI.   Lines 85-86: ADD the abbreviation for “sub-Saharan Africa in brackets so that it will read as follows ““……… therapy programmes in sub-Saharan Africa (SSA) [24].””   Line 95: replace “low- and middle-income countries” with its abbreviation LMICs   Lines 95-96: replace “quality improvement” with its abbreviation QI.   Line 96: replace “primary health care” with its abbreviation PHC. | Added abbreviation LMICs in brackets.  Replaced with abbreviation LMICs.  Replaced with abbreviation LMICs.  Replaced with abbreviation PHC.  Replaced with abbreviation PHC.  Replaced with abbreviation QI.  Replaced with abbreviation QI.  Added abbreviation SSA in brackets.  Replaced with abbreviation LMICs.  Replaced with abbreviation QI.  Replaced with abbreviation PHC. |
| 97 Review aim and questions   Line 98: replace “quality improvement (QI)” with its abbreviation – QI.   Line 99: replace “primary health care” with its abbreviation PHC.   Replace “low- and middle- income countries (LMICs)” with its abbreviation LMICs.   Line 101: replace “primary health care” with its abbreviation PHC.   Lines 101-102: replace “low- and middle- income countries” with its abbreviation LMICs.   Line 104: replace “primary health care” with its abbreviation PHC.   Lines 106-107: replace primary health care” with its abbreviation PHC. | Replaced with abbreviation QI.  Replaced with abbreviation PHC.  Replaced with abbreviation LMICs.  Replaced with abbreviation PHC.  Replaced with abbreviation LMICs.  Replaced with abbreviation PHC.  Replaced with abbreviation PHC. |
| 108 Review approach and methods  109 Review approach   Lines 123-124: replace “quality improvement” with its abbreviation QI.   Line 124: replace “primary health care” with its abbreviation PHC; and replace “low- and middle- income countries” with its abbreviation LMICs. | Replaced with abbreviation QI.  Replaced with abbreviation PHC; and LMICs, respectively. |
| 192 Data analysis   Line 193: replace “Quality Improvement (QI)” with its abbreviation QI.   Line 194: since the MUSIQ has already been written in long form in lines 87-88, I suggest to the authors to rephrase the sentence “The Model for Understanding Success in Quality (MUSIQ) developed by” to read as follows: The MUSIQ model developed by [27] was…….   Line 213: replace “primary health care” with its abbreviation PHC; and replace “quality improvement” with its abbreviation QI. | Replaced with abbreviation QI  Sentence re-phrased as advised.  Replaced with abbreviations PHC and QI, respectively. |
| 218 Results  219 Characteristics of included studies   Line 221: replace “primary health care” with its abbreviation PHC;   Line 222: replace “quality improvement” with its abbreviation QI.   Line 226: replace low- and middle-income countries (LMICs) with its abbreviation LMICs; and replace “quality improvement” with its abbreviation QI.   Line 227: replace “primary health care” with its abbreviation PHC.   Line 233: There seems to be an error OR a duplication of references. The references [41, 42] are not part of Table 2. Probably, the Authors meant References [71,72] which seem to be the same. Therefore, I suggest to the Authors to check on these references [41, 42] & [71, 74] both in the text and in the Reference List to ensure there is no any duplication. | Replaced with abbreviation PHC.  Replaced with abbreviation QI.  Replaced with abbreviation LMCI and QI, respectively.  Replaced with abbreviation PHC.  Checked and corrected, removed duplicated references 71&74 in text and in Reference List. |
| 275 Themes   Lines 276: replace “quality improvement” with its abbreviation QI; and replace “primary health care” with its abbreviation PHC.   Lines 277-278: replace the sentence “model for understanding success in quality (MUSIQ) and the consolidated framework for implementation research (CFIR),” with the following “"MUSIQ model and the CFIR"” | Replaced with abbreviations QI and PHC, respectively.  Sentence re-phrased. |
| 280 Theme 1: Microsystem and individual health worker(s) motivation   Line 291: replace “quality improvement” with its abbreviation QI.   Line 293: replace “primary health care” with its abbreviation PHC.   Line 299: DELETE “that” between workers & perceived.   Lines 341-342: replace “primary health care (PHC)” with its abbreviation PHC. | Replaced.  Replaced.  Deleted.  Replaced. |
| 358 Theme 2: Attributes of quality improvement intervention   Line 359: replace “quality improvement” with its abbreviation QI.   Line 370: since “sub-Saharan Africa” has been mentioned in lines 84-85 with suggestion to ADD its abbreviation in brackets (SSA); and also, in line 227 & line 285 I suggested to REPLACE it with its abbreviation. Therefore, here (370) it is also recommended to replace “sub-Saharan Africa (SSA)” with its abbreviation SSA.   Line 377: replace “primary health care” with its abbreviation PHC.   Line 378: replace “Quality improvement” with its abbreviation QI.   Line 390: replace “quality improvement” with its abbreviation QI.   Line 403: replace “quality improvement” with its abbreviation QI.   Lines 404-405: since “maternal and perinatal death surveillance and response (MPDSR)” is well written and abbreviated in Table 2 in the row containing Ref. 62 (Kinney et al.[62]), I suggest to the Authors to replace “maternal and perinatal death surveillance and response (MPDSR)” with its abbreviation MPDSR. | Replaced.  Replaced.  Replaced.  Replaced.  Replaced.  Replaced.  Replaced. |
| 428 Theme 3: Organization and 428 implementing team   Lines 432-433: replace “primary health care” with its abbreviation PHC.   Line 435: replace “primary health care” with its abbreviation PHC & quality improvement” with its abbreviation QI so that the line 435 will read as follows “Studies outlined the barriers to and enablers of PHC QI at”………   Line 436: replace “Sub-Saharan Africa (SSA)” with its abbreviation SSA.   Line 442: replace “primary health care” with its abbreviation PHC.   Line 448: replace “primary health care” with its abbreviation PHC.   Line 462: EDIT the word “arent” by ADDING an apostrophe between “n” & “t” as follows “aren’t” adequate……   Line 470: replace “primary health care” with its abbreviation PHC.   Line 472: replace “quality improvement (QI)” with its abbreviation QI. | Replaced.  Replaced with respective abbreviations.  Replaced.  Replaced.  Replaced.  Edited to read “aren’t”.  Replaced.  Replaced. |
| 526 Theme 4: Health systems support 526 and capacity   Lines 527-528: replace “primary health care (PHC)” with its abbreviation PHC.   Line 529: replace “primary health care (PHC)” with its abbreviation PHC.   Line 530: replace “quality improvement” with its abbreviation QI.   Line 540: replace “Sub-Saharan Africa” with its abbreviation SSA.   Line 544: replace “primary health care” with its abbreviation PHC.   Line 546: replace “quality improvement” with its abbreviation QI.   Lines 556-557: replace “quality improvement” with its abbreviation QI.   Line 559: replace “primary health care” with its abbreviation PHC.   Line 562: replace “primary health care” with its abbreviation PHC.   Lines 562-563: replace “low- and middle-income countries (LMICs)” with its abbreviation LMICs.   Line 565: replace “quality improvement” with its abbreviation QI.   Line 577: replace “quality improvement” with its abbreviation QI.   Lines 584-585: replace “primary health care” with its abbreviation PHC.   Line 587: replace “quality improvement” with its abbreviation QI.   Lines 591-592: replace “quality improvement” with its abbreviation QI. | Replaced.  Replaced.  Replaced.  Replaced.  Replaced.  Replaced.  Replaced.  Replaced.  Replaced.  Replaced.  Replaced.  Replaced.  Replaced.  Replaced.  Replaced. |
| 601 Theme 5: External environment and structural factors   Line 602: replace “quality improvement” with its abbreviation QI.   Line 614: replace Sub-Saharan Africa (SSA)” with its abbreviation SSA.   Line 621: EDIT the word "reportedly" to be reported & INSERT “as" between “reported & an” so that it will read as follows: “““Tajikistan, and its high-level prioritization by the central government, was reported as an”””   Line 625: replace “Primary Health Care” with its abbreviation PHC.   Line 632: replace “maternal perinatal death surveillance and response (MPDSR)” with its abbreviation MPDSR.   Line 643: replace “quality improvement” with its abbreviation QI; & replace “primary health care” with its abbreviation PHC.   Line 659: replace “quality improvement” with its abbreviation QI. | Replaced.  Replaced.  Edited to read “… reported as an…”  Replaced.  Replaced.  Replaced.  Replaced. |
| 671: Theme 6: Execution of quality improvement intervention   Line 672: replace “quality improvement” with its abbreviation QI.   Lines 674-675: replace “quality improvement” with its abbreviation QI.   Line 675: replace “primary health care” with its abbreviation PHC; and replace “low- and middle-income countries” with its abbreviation (LMICs).   Line 679: replace “Sub-Saharan Africa” with its abbreviation SSA.   Line 694: replace “quality improvement” with its abbreviation QI.   Line 709” EDIT the word “support” to be supportive so that the line will read as follows: ““Non-implementation of supportive supervision and limited training for implementers was””””.   Line 726: replace “primary health care” with its abbreviation PHC. | Replaced.  Replaced.  Replaced.  Replaced.  Replaced.  Replaced.  Replaced.  Replaced. |
| 748 Discussion   Lines 749-750: replace “quality improvement” with its abbreviation QI.   Line 750: replace “primary health care” with its abbreviation PHC; and replace “low- and middle-income country” with the abbreviation LMIC.   Line 752: replace “quality improvement” with its abbreviation QI.   Line 754: replace “quality improvement” with its abbreviation QI.   Line 759: replace “quality improvement” with its abbreviation QI.   Line 760: replace “primary health care” with its abbreviation PHC.   Line 765: replace “quality improvement” with its abbreviation QI.   Line 766: replace “primary health care” with its abbreviation PHC.   Line 770: replace “quality improvement” with its abbreviation QI.   Line 772: replace “quality improvement” with its abbreviation QI; and replace “primary health care” with its abbreviation PHC.   Line 774: replace “World Health Organization (WHO)” with its abbreviation WHO.   Line 776: replace “quality improvement” with its abbreviation QI.   Line 778: replace “primary health care” with its abbreviation PHC.   Lines 785-786: replace “Model for Understanding Success in Quality, MUSIQ” with MUSIQ model [27]   Lines 786-787: DELETE “Consolidated Framework for Implementation Research” and KEEP only the abbreviation CFIR.   Line 789: replace “primary health care” with its abbreviation PHC.   Line 803: replace “sub-Saharan Africa (SSA)” with its abbreviation SSA.   Line 812: replace “quality improvement” with its abbreviation QI.   Lines 815-816: replace sub-Saharan Africa” with its abbreviation SSA.   Lines 817-818: replace “maternal and perinatal death surveillance and response (MPDSR)” with its abbreviation MPDSR.   Line 827: replace “continuous quality improvement” with its abbreviation CQI; & replace quality improvement collaboratives” with its abbreviation QICs.   Line 828: replace “primary health care” with its abbreviation PHC.   Lines 841-842: replace “quality improvement” with its abbreviation QI.   Line 842: replace “primary health care” with its abbreviation PHC. | Replaced.  Replaced.  Replaced.  Replaced.  Replaced.  Replaced.  Replaced.  Replaced.  Replaced.  Replaced.  Replaced.  Replaced.  Replaced.  Replaced.  Replaced.  Replaced.  Replaced.  Replaced.  Replaced.  Replaced.  Replaced.  Replaced.  Replaced.  Replaced.  Replaced.  Replaced.  Replaced. |
| 857 Strength and limitations   Line 868: replace “quality improvement” with its abbreviation QI; and replace “primary health care” with its abbreviation PHC.   Line 870: replace “sub-Saharan Africa” with its abbreviation SSA. | Replaced.  Replaced. |
| 884 Conclusion   Line 885: replace “quality improvement” with its abbreviation QI.   Lines 885-886: replace “low- and middle-income countries” with its abbreviation LMICs.   Line 886: replace “primary health care” with its abbreviation PHC.   Line 897” replace “primary health care” with its abbreviation PHC. | Replaced.  Replaced.  Replaced.  Replaced. |
| 915 References  I suggest to the authors to re-check and ensure that REFs 41 & 42 are not duplicated in REFs 71 and 74.   Lines 1019-1025 (REF. 41 & 42):  1019 41. Baker U, Petro A, Marchant T, Peterson S, Manzi F, Bergström A, et al. Health workers'  1020 experiences of collaborative quality improvement for maternal and newborn care in rural Tanzanian  1021 health facilities: A process evaluation using the integrated 'Promoting Action on Research  1022 Implementation in Health Services' framework. PloS one. 2018;13(12):e0209092.  1023 42. Tancred T, Mandu R, Hanson C, Okuga M, Manzi F, Peterson S, et al. How people- centred health  1024 systems can reach the grassroots: experiences implementing community-level quality improvement in  1025 rural Tanzania and Uganda. Health policy and planning. 2018;33(1):e1-e13.   Lines 1111–1114 (REF 71) ; & Lines 1121-1123 (REF 74)  1111 71. Baker U, Petro A, Marchant T, Peterson S, Manzi F, Bergstrom A, et al. Health workers'  1112 experiences of collaborative quality improvement for maternal and newborn care in rural Tanzanian  1113 health facilities: A process evaluation using the integrated 'Promoting Action on Research  1114 Implementation in Health Services' framework. PLoS One. 2018;13(12):e0209092.  1121 74. Tancred T, Mandu R, Hanson C, Okuga M, Manzi F, Peterson S, et al. How people-centred health  1122 systems can reach the grassroots: experiences implementing community-level quality improvement in  1123 rural Tanzania and Uganda. Health Policy Plan. 2018;33(1):e1-e13. | Checked; removed duplicated references 71 & 74).  Checked; removed duplicated references 71 & 74). |
| *Part C: Reviewer #2* |  |
| The authors of this study conducted a systematic review of literature on the barriers and enablers of quality improvement in primary healthcare in low- and middle-income countries (LMICs). The study notion is interesting and it is well designed to answer the research objectives. However, the manuscript and some parts of the material need revision to make them publishable. My comments and suggestions in this regard are provided below. |  |
| 1. General: the text throughout the manuscript needs a language and grammar edit by a professional. | All suggested (grammar/language) edits by Reviewers have been addressed. |
| 2. Title and study design: although authors present this research output as a systematic review, it seems “scoping review” is a better title for this paper. I would like to refer the authors to a related publication about differences of these two types of review: https://link.springer.com/article/10.1186/s12874-018-0611-x, PMID: 30453902. | The authors thank the reviewer for highlighting this and sharing the insightful publication on this topic (Munn et al, 2018). Indeed, Munn and colleagues confirm that there are multiple and significant overlaps between scoping reviews and systematic reviews.  A checklist provided by Munn et al report that scoping reviews are indicated:  To identify the types of available evidence in a given field  To clarify key concepts/ definitions in the literature  To examine how research is conducted on a certain topic or field  To identify key characteristics or factors related to a concept  As a precursor to a systematic review  To identify and analyze knowledge gaps.  The authors do not see much alignment between the indications listed here and the present review’s aims. We did not seek to identify the type of evidence available on QI in PHC although the review process inevitably laid this bare. Note that we did not seek out all or any study on the topic, but rather those that would help answer the review question. The authors did not set out to identify key concepts or definitions, or to examine how research on QI is conducted, although again, inevitably, a passing note is made in the review regarding some theoretical frameworks used in research on PHC QI research but this was not a primary review question). Further, concepts used in the review were pre-existing, having been identified prior by MUSIQ, CFIR authors and others. The review was also not conducted as a precursor to a future systematic review. The two areas of alignment between the present review and scoping reviews (according to Munn et al) are to identify factors related to the topic and knowledge gaps (but these are not necessarily exclusively applicable to scoping reviews alone). Systematic reviews commonly identify such factors and or evidence blind spots).  On the other hand, table 1 (Munn et al) listing some six comparative characteristics of scoping reviews and reviews shows that the current study aligns very well with Munn and colleagues’ conceptualization of how a systematic review is designed, conducted ad reported with the single exception that Munn et al would prefer quantitative analysis/synthesis in the form of meta-analysis or meta-synthesis to summarize/combine included studies. This is not always possible especially where studies have disparate methods and vary in the reporting of findings, Besides, this requirement would exclude reviews incorporating qualitative and mixed methods.  Lastly, Munn and colleagues make an important note, that characteristics and conceptualization of both scoping reviews and systematic reviews is an evolving matter (as with many things knowledge/evidence), and it would be difficult to see or interpret their listed characteristics as cast in stone or a final verdict.  The authors wish to retain “Systematic Review” in the title given that we reviewed “a clearly formulated question using explicit, systematic methods to identify, select, and critically appraise relevant research, and to collect and analyze data from the studies that are included in the review” but remain open to receiving further guidance. |
| 3. Style: please format the study style and organization and headings of manuscript based the journal requirements: https://journals.plos.org/globalpublichealth/s/submission-guidelines#loc-systematic-reviews-and-meta-analyses | Study style and headings of manuscript have been checked and aligned with journal requirements. File format, length, font, layout and spacing, headings, language, abbreviations (factoring in feedback from Reviewer #1), figures and supporting information double-checked. |
| 4. Introduction: this section lacks an appropriate opening on the LMICs and why addressing the study notion in these countries is important. | An appropriate opening statement has been inserted and the section re-phrased. |
| 5. Methods: the abstract mentions that four authors were involved in the process of title screening to data extraction and synthesis; however, the study has only three authors in this submission. Besides, reading the methods section thoroughly, shows different stages of review and data extraction were done by different authors and not by all of them. For example, the data extraction is done by one and half of the citations were re-checked by another author. As we know a rigorous systematic review needs incorporation of at least two independent reviewers screening the citations and extracting the data. Please revise the methods section in abstract and full text to clarify these points and clearly provide how many authors were involved in each stage of study. | Although four reviewers participated in the review, the reviewers determined that one reviewer did not fully meet the four ICMJE requirements to be included as an author. Their contribution is instead acknowledged.  To remove any ambiguity, the relevant sections under Methods have been revised and harmonized. |
| 6. Methods: the last part of this section entitled “Data analysis” might be better to be renamed to “Data analysis and synthesis”. | Section has been renamed to read “Data analysis and synthesis”. |
| 7. Results: regarding the six themes extracted from studies through the review, it is suggested to add a figure summarizing the key points of each theme so a brief summary of the findings could be reviewed by the audience of this study. | Added a figure (Fig. 2) with a summary of key point for each theme |
| 8. Discussion: it is highly suggested that authors include a paragraph about how the findings of this study may help health authorities and policy makers via the extracted themes to improve the quality of primary healthcare in LIMCs. | A paragraph has been inserted 778-794 in marked up copy (749-765 in clean copy), teasing out the implications of the study for policymakers. |
| 9. Supporting information and material could be appended into one file with a dedicated index page, for an easier access and use. | Where possible, supporting information has been appended into one file. However, those with varied or incongruent formats are hard to combine and may be uploaded/availed separately. |
